# Effect of integrating maternal and child health services, nutrition and family planning services on postpartum family planning uptake at 6 months post-partum in Burkina Faso, Cote d’Ivoire and Niger: a quasi-experimental study protocol

**DOI:** 10.1186/s12978-022-01467-x

**Published:** 2022-08-20

**Authors:** Denise Kpebo, Abou Coulibaly, Wambi Maurice Evariste Yameogo, Sujata Bijou, Ramatoulaye Hamidou Lazoumar, Halima Tougri, Marguerite N’dour, Seni Kouanda

**Affiliations:** 1 Unité de Formation et de Recherche en Sciences Médicales d’Abidjan (DSPIM-UFR/SMA), Université Félix Houphouet Boigny, 01 BPV 34, Abidjan 01, Côte d’Ivoire; 2 Institut Africain Santé Publique (IASP), 12 BP 199, Ouagadougou, Burkina Faso; 3 Institut de recherche en sciences de la santé (IRSS), 03 BP 7047, Ouagadougou 03, Burkina Faso; 4 IntraHealth International, 6340 Quadrangle Drive, Suite 200, Chapel Hill, NC 27517 USA; 5 Centre de Recherche Médicale et Sanitaire, 634, Boulevard de la Nation, YN034, BP 10887, Niamey, Niger; 6 IntraHealth International, Sect. 15 Ouaga 2000, Ouagadougou, Burkina Faso

**Keywords:** Maternal and infant health, Postpartum famly planning, Integration of services, Maternal-Child Health Services/organization & administration, Santé maternelle et infantile, Planification familiale post-partum, Intégration des services, Services de santé maternelle et infantile/organisation et administration

## Abstract

**Background:**

Although several interventions integrating maternal, neonatal, child health and nutrition with family planning have been implemented and tested, there is still limited evidence on their effectiveness to guide program efforts and policy action on health services integration. This study aims to assess the effectiveness of a service delivery model integrating maternal and child health services, nutrition and family planning services, compared with the general standard of care in Burkina Faso, Cote d'Ivoire, and Niger.

**Methods:**

This is a quasi experimental study with one intervention group and one control group of 3 to 4 health facilities in each country. Each facility was matched to a control facility of the same level of care that had similar coverage on selected reproductive health indicators such as family planning and post-partum family planning. The study participants are pregnant women (up to 28 weeks of gestational age) coming for their first antenatal care visit. They will be followed up to 6 months after childbirth, and will be interviewed at each antenatal visit and also during visits for infant vaccines. The analyzes will be carried out by intention to treat, using generalized linear models (binomial log or log Poisson) to assess the effect of the intervention on the ratio of contraceptive use prevalence between the two groups of the study at a significance level of 5%, while taking into account the cluster effect and adjusting for potential confounding factors (socio-demographic characteristics of women unevenly distributed at inclusion).

**Discussion:**

This longitudinal study, with the provision of family planning services integrated into the whole maternal care continuum, a sufficiently long observation time and repeated measurements, will make it possible to better understand the timeline and the factors influencing women’s decision-making on the use of post-partum family planning services. The results will help to increase the body of knowledge regarding the impact of maternal and child health services integration on the utilization of post-partum family planning taking into account the specific context of sub-Saharan Africa French speaking countries where such information is very needed.

## Plain Language Summary

One strategy to improve the utilization of health services by mothers and their children is the integration of maternal and child health services. For instance, a pregnant woman coming for an antenatal care visit would also receive counseling on post-partum family planning services and maternal nutrition. Similarly, a woman coming for her infant’s vaccines would be offered counseling on post-partum family planning, maternal nutrition and breastfeeding. Although several interventions have been implemented and tested, there is still limited evidence on the conditions and factors required for successful maternal and child health services integration strategies. This study aims to assess the effectiveness of an intervention integrating maternal and child health services, nutrition and family planning services. For the purpose of the evaluation, 2 distincts groups of health facilities will be selected in each country, one group of 3 or 4 health facilities where the intervention will be implemented, and another group of 3 or 4 health facilities with the general standard of care. The study participants are pregnant women (up to 28 weeks of gestational age), coming for their first antenatal care visit, who will be followed up to 6 months after childbirth.The analyzes will be carried out to assess the effect of the intervention on contraceptive use prevalence between the two groups of health facilities. This study will make it possible to better understand the timeline and the factors influencing women’s decision-making on the use of post-partum family planning services.

## Résumé

**Contexte:**

Bien que plusieurs interventions d’intégration des services de santé maternelle, néonatale et infantile, de nutrition, et de planification familiale aient été déjà mises en œuvre et évaluées, il existe encore peu de preuves de leur efficacité pour guider les efforts des programmes et des politiques visant l'intégration des dits services de santé. Cette étude vise à évaluer l'efficacité d'un modèle de prestation de services intégrant les services de santé maternelle et infantile, les services de nutrition et de planification familiale, en comparaison des prestations standards de soins au Burkina Faso, en Côte d'Ivoire et au Niger.

**Matériels et méthodes:**

Il s'agit d'une étude quasi expérimentale avec un groupe d'intervention et un groupe témoin de 3 à 4 établissements de santé par pays. Chaque établissement a été apparié à un établissement témoin de même niveau qui avait une couverture similaire sur certains indicateurs de santé reproductive tels que la planification familiale et la planification familiale du post-partum. Les participantes à l'étude sont des femmes enceintes (jusqu'à 28 semaines d'âge gestationnel) venant pour leur première consultation prénatale. Elles seront suivies jusqu'à 6 mois après l'accouchement, et seront interrogées à chaque consultation prénatale et également lors des visites de vaccination des nourrissons. Les analyses seront réalisées en intention de traiter, à l'aide de modèles linéaires généralisés (binomial log ou log Poisson) pour évaluer l'effet de l'intervention sur le ratio de prévalence de l'utilisation de la contraception entre les deux groupes de l'étude à un seuil de significativité de 5%, tout en tenant compte de l'effet de grappe et en ajustant les facteurs de confusion potentiels (caractéristiques socio-démographiques des femmes inégalement réparties à l'inclusion).

**Discussion:**

Cette étude longitudinale, avec une offre de services de planification familiale intégrée à l'ensemble du continuum de soins maternels, un temps d'observation suffisamment long et des mesures répétées, permettra de mieux comprendre la chronologie et les facteurs influençant la prise de décision des femmes sur l'utilisation des services de planification familiale post-partum. Les résultats contribueront à accroître le corpus de connaissances sur l'impact de l’intégration des services de santé maternelle et infantile sur l'utilisation de la planification familiale post-partum en tenant compte du contexte spécifique des pays francophones d'Afrique subsaharienne où de telles informations sont plus que nécessaires.

## Background

Post-partum Family Planning (PPFP) is recognized as a key intervention in reducing maternal and child mortality [1, 2]. Indeed, pregnancies during the first year postpartum are the most risky because of increased risks of miscarriage, abortion, postpartum hemorrhage and anemia for the mother, and preterm birth, stillbirth, and newborn death for the child [3].

Family Planning (FP) programs, by allowing optimal birth spacing, have effectively contributed to a significant reduction of 32% of all maternal deaths and 10% of all child deaths [1, 2].

Yet, unmet contraceptive need is high for many postpartum women in sub-Saharan Africa (SSA). A study conducted in 21 low- and middle-income countries showed that an estimated 61% of postpartum women had unmet contraceptive needs [4]. Furthermore, research indicates that about 40% of women state they intend to use a contraceptive method in the first year of postpartum but do not do so [5].

To reduce unmet contraceptive need, postpartum women need access to family planning information and services. However, many women in SSA do not attend health facilities in the post-partum period and even fewer go for addressing specific PPFP needs [3]. However, these women’s contacts with the health systems are more frequents when considering the entire period going from pregnancy to post-partum. In SSA, more than 75% of pregnant women will benefit at least 1 ANC during their pregnancy and most postpartum women do seek routine health services for their infants, including for immunizations [4, 6]. Thus, the pregnancy to post-partum period provides an important opportunity to reach women repeatedly with PPFP messages and services.

Evidence shows that integrating FP with maternal, neonatal, and child health (MNCH) is a key strategy for improving maternal and child health care indicators [7, 8]. For instance, in Malawi such service integration resulted in a 14% increase in the uptake of FP methods [9]. Similarly, in Liberia and Rwanda, the integration of FP with immunization resulted in an increase uptake of PPFP methods [10, 11]. Another recent study in Ethiopia examining the integration of FP with MNCH showed a 6% increase in the uptake of FP services [12]. In addition, studies showed that integrating MNCH and FP services would cost approximately $1.5 billion less than providing MNCH services alone [13]. Despite these proven benefits, integration of health services remains weak, especially in SSA countries [14]. While many national policy recommandations support health service integration, this is not effective at the delivery point of contact [14].

Maternal and child mortality remains major public health issues in SSA and the situation is particularly alarming in the nine French speaking countries of the Ouagadougou partnership in western SSA. It is estimated that one in 41 women die from maternal causes in these countries, compared to 1 in 54 in developping countries, and 1 in 4900 in developed countries [15]. Neonatal and child mortality rates are high and modern contraceptive prevalence rate (mCPR) are low. For instance in Niger, mCPR is 11% among married women while unmet need for FP is 39.2% in Cote d’lvoire [15].

Although several integrated service delivery initiatives targeting FP and MNCH have been implemented and tested [16–20], there is still limited evidence to support such interventions’ effectiveness. Indeed, most of these interventions presented shortcomings not only at the conceptual level but also in terms of the methodology used [10]. In general, these integration initiatives rarely involved more than two services, and nutriton services were generally poorly included among the services offered [21–23]. The evaluations of the effects of these interventions focused mainly on the coverage of services [10, 13, 18]. Finally, the evaluation design used are not always robust; most of them being mere before and after study design with no control group [17, 24].

In order to fill these gaps in, we have developed this protocol for a longitudinal quasi experimental study. The general objective of this study is to assess the effectiveness of a service delivery model integrating maternal and child health services, nutrition, and family planning services, compared with the general standard of care in Burkina Faso, Cote d'Ivoire, and Niger. Specifically, this study aims at (i) assessing the effects of the intervention on the uptake of post-partum family planning (PPFP) at 6 months post-partum, (ii) assessing the effects of the intervention on the use of integrated health services, (iii) and assessing the effects of the intervention on improving maternal and neonatal health indicators during pregnancy and the immediate postpartum period.

## Methods and study design

### Study settings, sites and study design

Burkina Faso, Cote d'Ivoire, and Niger are 3 West african French speaking countries with poor level of maternal and child health indicators. Some information related to relevant reproductive health and health service utilization indicators based on demographic and health survey data from theses countries, Burkina Faso (2010), Cote d’Ivoire (2011–2012) and Niger (2012) are presented in Table 1.

**Table 1:** Selected indicators on reproductive health and utilization of maternal and child health services Burkina Faso (2010), Cote d’Ivoire (2011–2012) and Niger (2012)

Selected indicators	Burkina Faso	Cote d’ Ivoire	Niger
Maternal mortality rate	330 per 100,000 live births in 2015	614 per 100,000 live births in 2015	520 pour 100,000 live births in 2015
Neonatal mortality rate	23 per1000	33 per 1000	28 per 1000
Child mortality rate	42 per 1000	27 per 1000	48 per 1000
mCPR	22.5%	23.5%	11%^a^
Unmet need of FP	23.3%	39.2%	15%^a^
Prevalence of acute malnutrition	8.6%	6%	10.3%
Prevalence of chronic malnutrition	21.2%	21.6%	42.2%
Prevalence of underweight	16.2%	12.8%	–
Early breastfeeding	56%	37%	–
Proportion of infants under 6 months of age who have benefited from exclusive breastfeeding	47.8%	23.5%	30%
Proportion of anemia in pregnant women	58%	64%	55.6%

^a^Among women in union

We will conduct a quasi experimental study in the selected countries health facilities. In each country, we will have one intervention group of facilities and one control group of facilities. Participants of the intervention group, meaning women attending the intervention facilities, will receive the full package of integrated PPFP/MNCH/Nutrition services, while participants in the control group will receive standard care. In each country, 3 to 4 health facilities have been identified as the intervention sites including a district hospital, an urban health center, and a rural health center. Each facility was matched to a control facility of the same level of care and that had similar coverage on selected reproductive health indicators such as family planning and post-partum family planning (Table 2). For each country, the selection of the intervention districts and health facilities was purposively made by a working group on Maternal, Neonatal and Child Health (MNCH) from the Ministry of Health.

**Table 2:** Sites of study

Country	Intervention settings	Control settings
Burkina Faso	• CMA of Po • CSPS niché at the CMA • CSPS of Tiébélé	• CMA of Kombissiri • CSPS niche at the CMA • CSPS de Toécé
Cote d’Ivoire	• HG district of Agnibilekro • CSU of Damé • CSR of Assuamé	• HG of Adzopé • CSU Assikoi • CSR Ananguié
Niger	• HD of Aguié • CSI urban of Aguié • CSI rural of Débi • CS of Zabon Moussou	• HD Guidanroumji • CSU urban of Guidanroumji • CSI rural of Karazome • CS of Tabouka

HD, CMA, and HG: General Hospital or Health District Hospital; CSPS, CSU, CSR, CSI: Primary Health care facilities (CSU = urban, CSR = rural)

### Description of the intervention

As part of the Ouagadougou partnership, the conferences held in Ouagadougou, Burkina Faso (2011) and London (2012) and the creation of FP2020 drew attention to FP in Francophone West African countries, which are lagging far behind other regions in terms of use of modern contraceptive methods [15]. Since then, notable progress has been made in these countries, including a 40% increase in the number of new FP users between 2011 and 2015 [15]. However, additional efforts are still needed for these countries to accelerate the use of essential quality FP and MNCH care [15].

The INSPiRE initiative is a project that aims to support the nine countries of the Ouagadougou Partnership to intensify their efforts by investing in the integration of FP and MNCH services. The vision of the INSPiRE Initiative is that all nine countries of the Ouagadougou Partnership achieve their national objectives in terms of increasing the modern contraceptive prevalence rate (mCPR); preventing maternal and child deaths and preventable diseases; improving maternal nutrition and infant and young child feeding practices, and improving the health, nutrition and well-being of newborns and infants. INSPiRE is based on the development and testing of models of excellence in the delivery of integrated MNH, PPFP and Nutrition services.

For the pilot phase of this project implementation, three countries have been selected: Burkina Faso, Cote d'Ivoire and Niger. The model of integrated health service delivery includes the community level (community contacts with community health workers), the intermediate level (rural and urban basic health centers), and the central level with the district hospital.

**Fig. 1:**
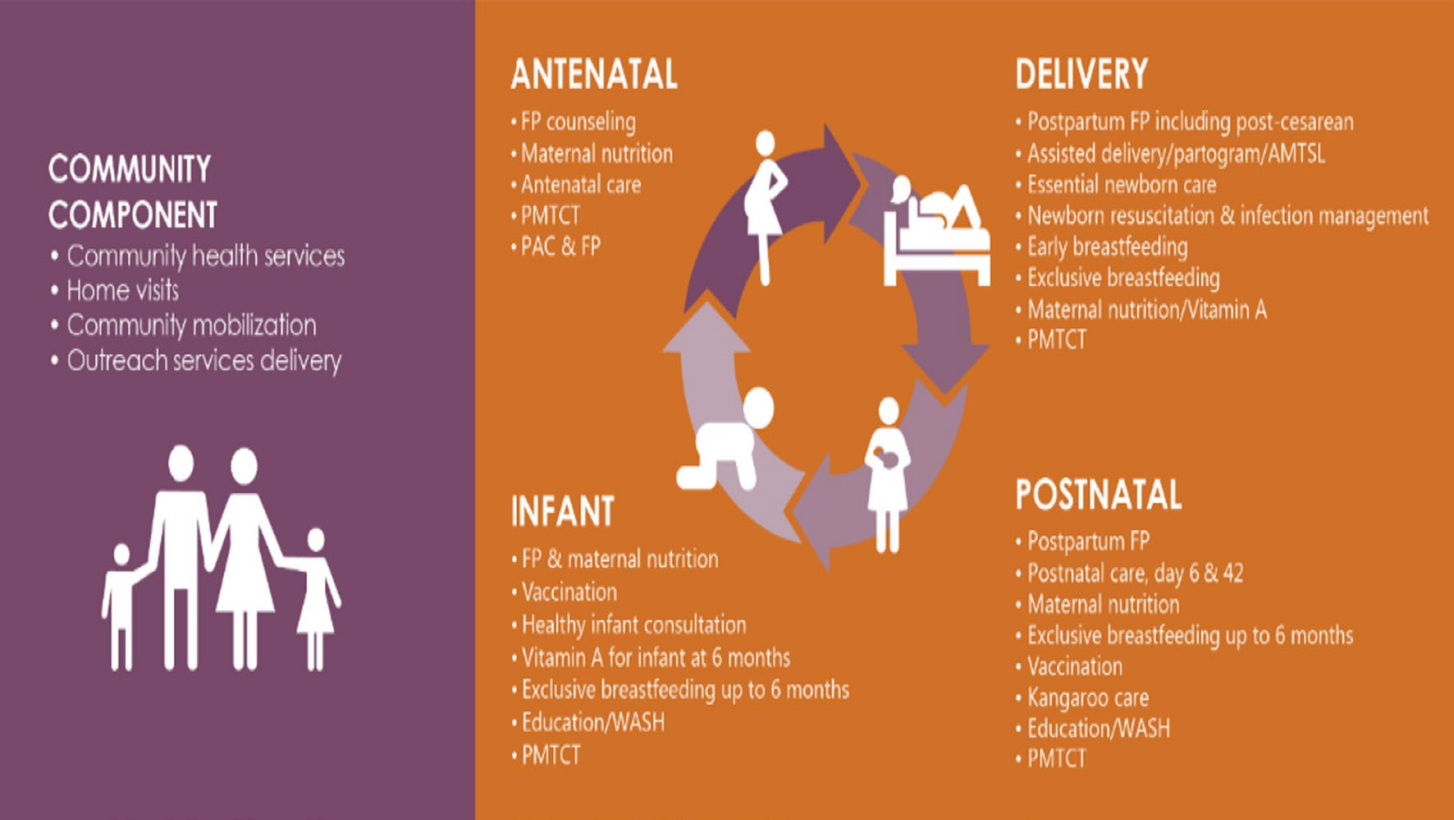
The intervention of integrated health services delivery

The standard model of integrated PPFP/MNCH/Nutrition service delivery has four points of contact where integrated services should be offered in the health facility: antenatal care, childbirth, postnatal care, and infant wellness visits (Fig. 1). In the model, these services are delivered during the same client visit based on client needs and standards of care. For instance, a pregnant woman would be offered PPFP counseling at each contact with the health system, along with counseling on other services such as maternal and infant nutrition.

### Study participants

Inclusion criteria

The study participants are pregnant women who meet the following criteria:

- Attending maternal health services for ANC;
- Having a pregnancy up to 28 weeks of gestational age;
- Being a resident of the communities served by the health facility;
- Not planning to travel for more than 1 month during pregnancy or for 6 months after childbirth;
- Having the intention from the outset to follow preventive care and childbirth in the health facility.

Exclusion criteria

The study participants will be excluded from the analysis if:

- They are lost to follow-up before giving birth;
- They experienced an abortion;
- They have refused to continue before giving birth.

### Participants recruitment process

Participants will be recruited at the ANC unit. All women coming for their first ANC visit will be invited to participate in the study. Women who accept will be screened according to the inclusion criteria. Those meeting the study criteria will be included in the study. In addition, each woman will have a unique identification number which will be entered in the registers to allow follow up throughout the duration of the study.

### Participant follow-up procedures

Women with a pregnancy up to 28 weeks of gestational age will be followed up to 6 months after childbirth, and will be interviewed at each antenatal visit and also during visits for infant vaccination, as detailed in the Table 3.

**Table 3:** Participants follow-up schedule

	ANC1	ANC2	ANC3	ANC4	ANC5	ANC6	ANC7	ANC8	Delivery	Early PNC Day 6–10	Late PNC Day 42–56	M2	M3	M4	M6
Admission	X														
Follow-up		X	X	X	X	X	X	X	X	X	X	X	X	X	X

### Outcomes measures

The primary outcome is the uptake of modern contraceptive methods at 6 months postpartum (proportion of women using modern contraceptives at 6 months in the experimental vs. control group).

Secondary outcomes are related to health indicators, services delivered and service utilization, as detailed in the Table 4, along with their definition and/or measurement process. Maternal infections will be considered for clinical signs of postpartum infection in mothers before discharge from hospital. Early neonatal infections will be considered as reported by health care providers in the patient file or based on obvious clinical signs of neonatal infection (fever, hypothermia, jaundice).

**Table 4:** Secondary outcomes of interest

Secondary outcomes of interest		
Health indicators	Definition/measurement	Sources
Early breastfeeding	Early initiation of breastfeeding, within one hour of birth, according to WHO definition [25]	Health facility registry or health card (as notified by health providers)
Birth weight	Baby’s weight measured using a baby scale	Health facility registry or health card (as notified by health providers)
Exclusive breastfeeding 0–6 months	The proportion of children, 0–6 months of age, fed only breastmilk, with the exception of oral rehydration solution, vitamins, minerals, and/or medicines [25]	Study participants surveys (as reported by study participants to data collectors)
Moderate acute malnutrition	A weight-for-height indicator between − 3 and − 2 z-scores (standard deviations) of the international standard or by a mid-upper arm circumference (MUAC) between 11 and 12.5 cm [25]	HF registry or health cards (as reported by providers
Severe acute malnutrition	Severe acute malnutrition is defined by a very low weight for height (below − 3z scores of the median WHO growth standards) [25]	HF registry or health cards (as reported by by provders
Vaccine coverage for children 0–6 months	Children from 0 to 6 months up to date with their vaccines	HF registry or health cards (as reported by health providers
Vitamin A supplementation and deworming		Health cards (as reported by providers)
Cough		Reported by study participants to data collectors
Diarrhea		Reported by study participants to data collectors
Malaria	Confirmed by a rapid diagnostic test or diagnostic by a health provider	Reported by study participants to data collectors
Maternal infections	Clinical signs of postpartum infection in mothers before discharge from hospital	Health facility registries as reported by providers
Neonatal infections	As reported by health care providers in the patient file or based on obvious clinical signs of neonatal infection (fever, hypothermia, jaundice)	Health facility registries as reported by providers or participants survey
Services delivered		
Number of individual FP counseling sessions during ANC		As notified by providers and collected by data collectors through out the study
Nutritional advice received during ANC		As notified by providers and collected by data collectors through out the study
Nutritional advice received during post- natala care		As notified by providers and collected by data collectors through out the study
Number of individual FP counseling sessions during ANC		As notified by providers and collected by data collectors through out the study
Services utilization		
Number of ANC visits (retention)		As notified by providers and collected by data collectors through out the study
Number of Post-partum visits		As notified by providers and collected by data collectors through out the study
Infant growth monitoring (weighing)		As notified by providers and collected by data collectors through out the study

### Sample size calculation

According to the Population Division of the United Nations Department of Economic and Social Affairs, the modern contraceptive prevalence among married or in union women in 2020 was estimated at 22.4% Cote d'Ivoire, 28.1% for Burkina Faso and 15.1% for Niger [26]. Considering an improvement of 15% in this proportion, attributable to the intervention, with a power of 80%, a significance level of 5%, and finally an intraclass correlation coefficient of 0.015, Cote d'Ivoire will have the largest sample size, about 88 women per cluster. By increasing the size by 15% to take into account any lost to follow-up related to travel, we’ll have a size of 102 women per health facility, meaning a total of 306 women per group in Cote d’Ivoire, and 268, and 172 per group in Burkina Faso and Niger, respectively. It is important to mention that the project health centers in each country were considered as clusters.And since the number of clusters is known in each country, we calculated the number of participants to be included so that the study have a good power. Details for each country are provided in the Table 5.

**Table 5:** Parameters used for sample size calculation

Caracteristics	Cote d’ivoire	Burkina Faso	Niger
Power	80%	80%	80%
Modern contraceptive prevalence rate	22.4%	28.1%	15.1%
Size of the expected effect in intervention groups	15.0%	15.0%	15.0%
Modern contraceptive prevalence rate (expected)	37.4%	43.1%	30.1%
Power	80%	80%	80%
Significance	5%	5%	5%
Intercluster correlation coefficient	0.015	0.015	0.015
Size per cluster	88	58	37
Size increased per cluster (15% increase)	102	67	43
Number of clusters per group	3	4	4
Final size of groups	306	268	172
Final size by country	612	536	344

### Data collection

Data will be collected through direct interview with health facility clients and extraction of data from health facility registers.

Direct interview with health facility clients: They will be carried out with the study participants at the various follow-up points. Data will be collected from a standardized questionnaire integrated into electronic tablets.Interviews will be carried out in health centers on the day of the woman’s consultation at her convenience. Otherwise, the interviewer will get in touch with the participant to agree on a day and place for the interview. In addition, these interviews will comply to barrier measures against Covid-19 (wearing a mask and a maintaining a distance of at least one meter between the investigator and the respondent).

Data extraction: Data will be extracted from health facilities registers (ANC, Childbirth, post-natal care, Immunization).

### Recruitment and training of investigators

Data will be collected by six females interviewers per country who speak the local languages.

They will be trained for 1 week on the study procedures and the content of the questionnaire.

### Data quality procedures

Data quality control procedures will be put in place to ensure that accurate data are recorded in the registers and entered into the database. Guidelines for data collection and the establishment of a registration register will be produced. In order to ensure that data will be collected in a standardized way in all participating health facilities, a pilot test of data collection and data management will be carried out before the beginning of baseline data collection. Data quality reports will be produced regularly for each health facility. Site control supervisions in the participating health facilities will be carried out regularly and a verification of the source data will be carried out to ensure that the data collected is accurate, complete, precise and reliable.

The supervisions will be carried out by the principal investigator and the country co-investigators.

### Data analysis

A statistical analysis plan will be developed. Descriptive statistics will be reported by calculating frequencies and percentages for categorical variables and means, standard deviations, and minimum and maximum values for the continuous variables if they are normally distributed.

Since the main outcome is contraceptive use, we will use generalized linear models (binomial log or log Poisson) to assess the effect of interventions on the ratio of contraceptive use prevalence between the two groups of the study at a significance level of 5%, while taking into account the cluster effect and adjusting for potential confounding factors (socio-demographic characteristics of women unevenly distributed at inclusion). All bi and multivariate analyzes will be carried out by intention to treat, including all women, whether or not they continued to visit health centers after inclusion. The unit of analysis will therefore be the woman. The analyzes will be carried out with Stata.

### Ethics

This protocol has been approved by the Institutional Review Committee of Intrahealth International as well as the respective ethical committees of the selected countries.

To ensure the safety and well-being of participants (healthcare providers and pregnant women) and to ensure no harm to them for this study, the team will take the following measures:

- All women meeting the inclusion criteria will be provided with detailed information on the objectives and procedures of the study and free and informed consent will be required prior to inclusion.
  - Pregnant women under 18 who are in union will be considered as emancipated minors and consent will be obtained directly as for an adult.
  - For pregnant women under the age of 18 who are not in a union, the consent of an adult parent/legal guardian will be required as well as the assent of the young girl.
- All research investigators and study staff will be trained to clearly communicate and perform the consent process.
  There will be no risk to women who decide not to participate in the study. Women who will not consent to participate in the study will receive the same care and access to services as those who have consented to participate in the study. If a condition warranting referral (including domestic violence, substance abuse, HIV counseling and testing, or any other relevant condition) is detected during the provision of antenatal care services (as part of this research), the study team will ensure that the woman is correctly referred and that the appropriate standard referral procedures are followed.
- The data collected on the tablets will be sent directly to the local IRSS-based server once a week. The server will be protected by a password known only by the Data manager. All research assistants and study staff will be trained to ensure data security.
  The published data will be depersonalized, described in a comprehensive manner, if possible and the anonymity of the participants will be preserved at all times.

### Study timeline

This study will last 18 months, from July 2021 to December 2022 as follows:

- 4 months for participants recruitment;
- 11 months of follow-up from the last woman included (she will be recruited during the first trimester of pregnancy at earliest, therefore 5-6 months of follow-up while she is still pregnant and 6 months of follow-up after delivery);
- 3 months for report writing.

## Discussion

Although significant interest in integrating family planning with other health services emerged during the last 30 years, both for programmatic and political reasons, limited empirical evidence is available on the effectiveness of programs that integrate family planning with maternal, perinatal, and child health [18]. Moreover, there is a paucity of evidence from developing countries in terms of what intervention programs work best for PPFP [13, 18, 27]. Of the relatively very few studies on integration that have been conducted, most were limited by methodological quality including cross-sectional design, hospital based surveys, non-family planning outcomes as main interest, and a short duration of observation [27, 28]. Overall, it is recognized that the evidence of the integration of postpartum family planning with other health services remains weak, and well-designed evaluation research is needed [13, 18, 27–29].

Previous studies showed that the results of the implact of integration of PPFP services into maternal health services on uptake of contraceptive methods are mixed. Some studies have found a relationship between ANC and contraceptive adoption in the post-partum but information was missing on whether or not PPFP counseling was provided during the ANC sessions [30, 31]. Other studies did not find any relationship between the integration of PPFP counseling into ANC and the uptake of contraceptive method in the postpartum period, but did rather find an impact of these services integration on women’s intention to use contraceptives methods [32]. However, integration of PPFP counseling into delivery care and postnatal care has shown more consistent positive impact on increasing adoption of PPFP [33–35].

As compared to these previous studies, one of the advantages of this intervention is that the provision of PPFP services will not be limited to just a few points of contact, but will rather be integrated into the whole maternal care continuum. In addition, this longitudinal study, with a sufficiently long observation time and repeated measurements, will make it possible to better appreciate the timeline and factors influencing women’s decision-making on the use of PPFP.

As for any multisite study, expected challenges regarding this protocol implementation include coordination issues with and between study countries and study sites, political and policy changes, eventual constraints in delivering the intervention with fidelity, and maintaining the intervention timelines [36]. To mitigate these threats, a technical working group is set up in each country to follow the overall implementation of the intervention, including the evaluation study. This technical working group is comprised of several stakeholders including those of the MOH to ensure the country engagement in the study process. In addition, a study coordination team is set up in each country to facilitate coordination between the different countries and study settings. Finally, a journal of events will be completed throughout study implementation to monitor any factor or event that could influence study outcomes so as to be considered during results analysis.

This protocol also has a number of strengths; primarily the longitudinal design will allow us to assess exposure to postpartum counseling and uptake of contraception at frequent intervals throughout the postpartum period limiting recall bias. As we will collect information on ongoing pregnancy and health services utilization, we will also limit confounding due to temporal changes that are present when including past births. We therefore believe that the overall strengths of the proposed design outweigh its limitations and the coming results will help to increase the body of knowledge on this topic, especially for SSA French speaking countries.

## Conclusion

This project will evaluate the impact of integrating FP with MNCH and nutrition services. Furthermore, this study will make it possible to better understand the timeline and factors influencing women's decision-making on the use of PPFP.

## Data Availability

Not applicable, no data or material was used at this stage of the study.

## Abbreviations

ANC: Antenatal care visit
CMA: Centre Médical avec antenne chirurgicale (Medical center with a surgical unit)
CS: Case de santé (health hut)
CSI urbain: Centre de Santé Intégré urbain (primary health care facility in urban area)
CSI rural: Centre de Santé Intégré rural (primary health care facility in rural area)
CSPS: Centre de santé et de promotion sociale (health and social promotion center)
CSU: Centre de santé urban (primary health care facility in urban area)
CSR: Centre de santé rural (primary health care facility in rural area)
FP: Family planning
HD: Hôpital de District (District level hospital)
HG: Hôpital General (General hospital or district level hospital)
MNCH: Maternal, neonatal, and child health services
MNCHN: Maternal, neonatal, child health services and nutrition
PPFP: Post-partum family planning
SSA: Sub-Sahara Africa
